# Supplementary material for: Organic Anion Transporters (OAT) and Other SLC22 Transporters in Progression of Renal Cell Carcinoma
Source: Cancers (Basel). 2022 Sep 29;14(19):4772. doi: 10.3390/cancers14194772 (PMC9563088; doi:10.3390/cancers14194772)
Supplement: Supplementary file 1 [file cancers-14-04772-s001.zip › cancers-1887853-supplementary/cancers-1887853-supplementary/supplementary Figures and Tables.pptx]

## Slide 1
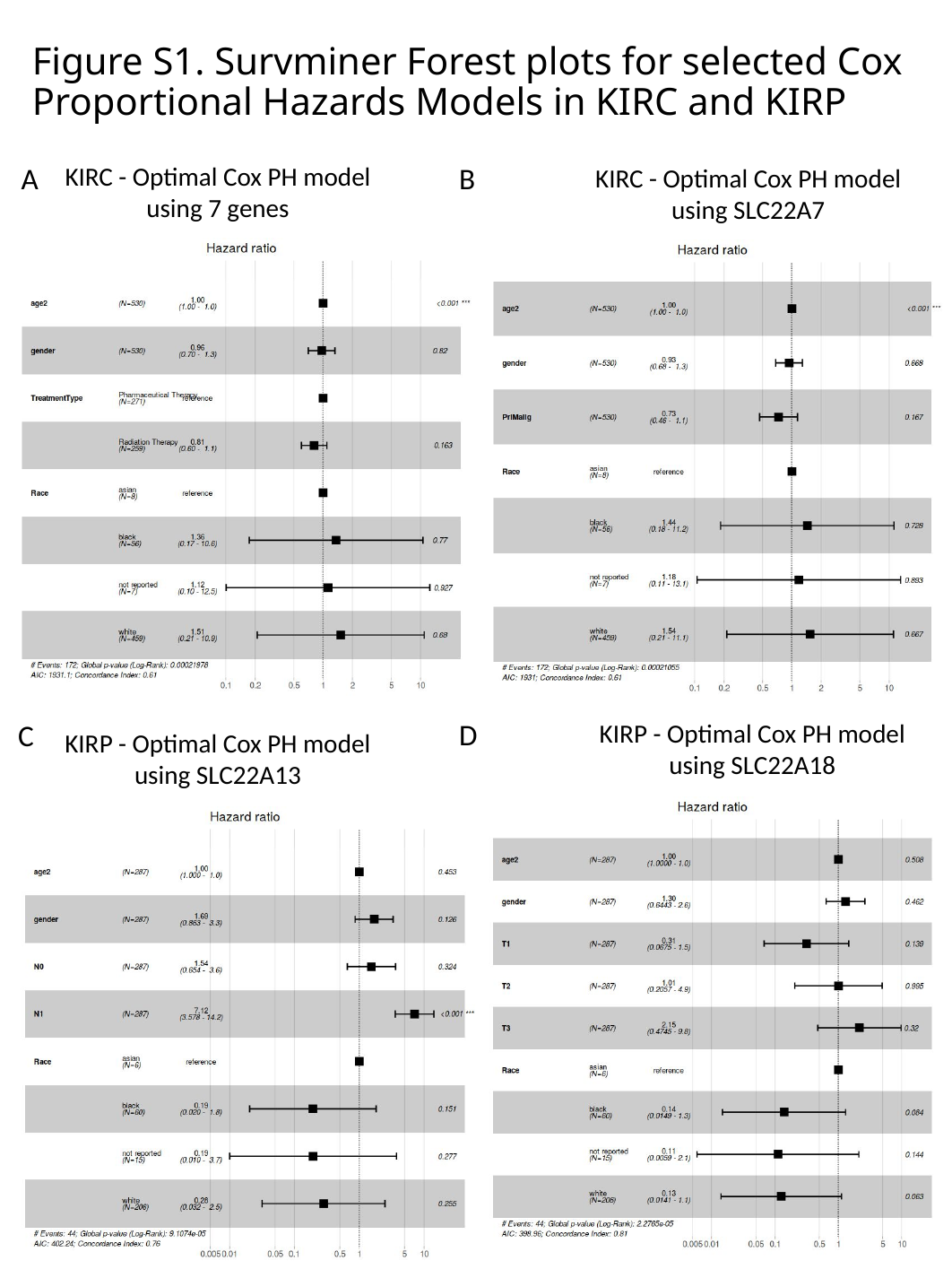

# Figure S1. Survminer Forest plots for selected Cox Proportional Hazards Models in KIRC and KIRP
A
B
KIRC - Optimal Cox PH model using 7 genes
KIRC - Optimal Cox PH model using SLC22A7
D
KIRP - Optimal Cox PH model using SLC22A18
C
KIRP - Optimal Cox PH model using SLC22A13

## Slide 2
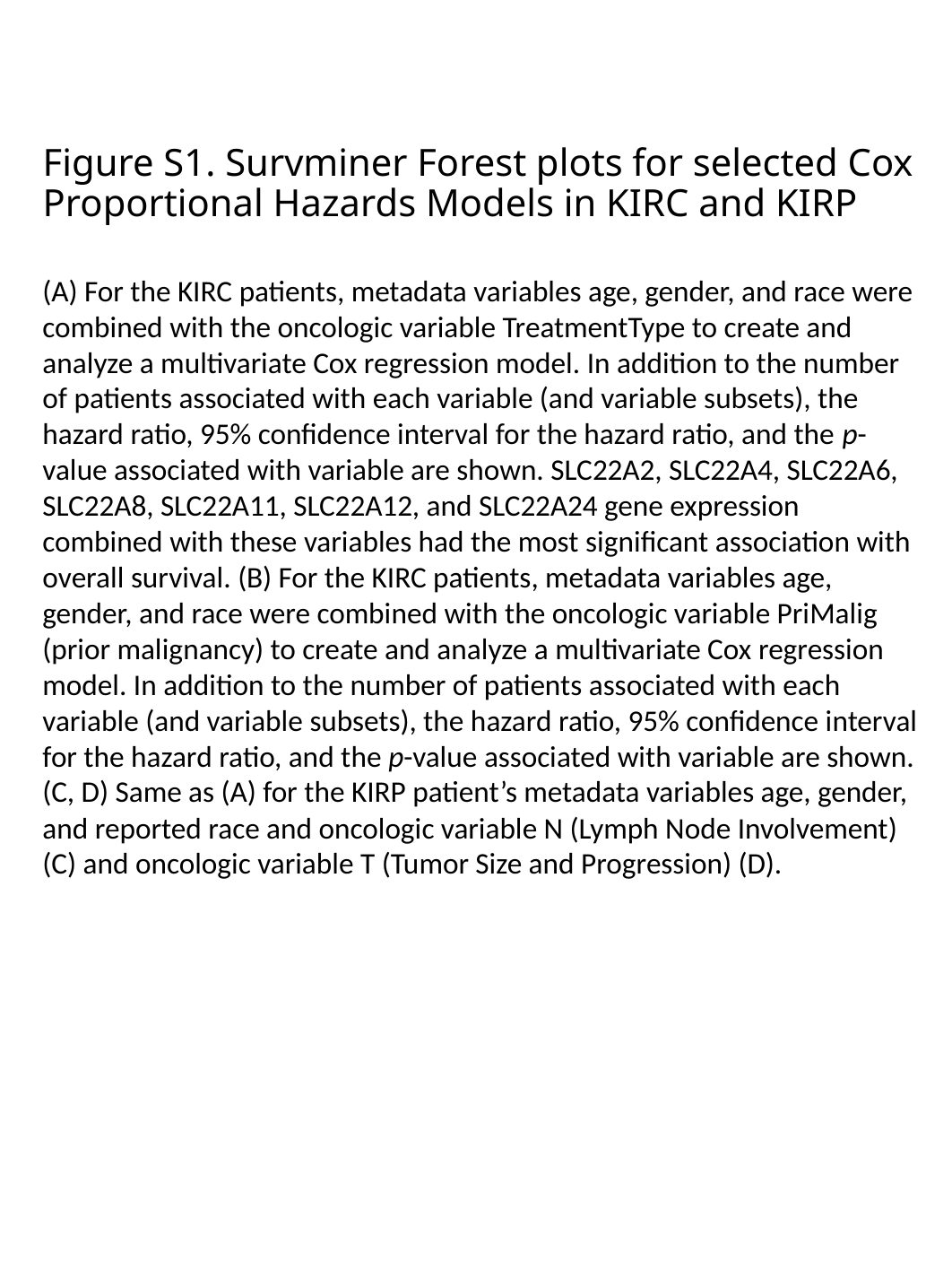

# Figure S1. Survminer Forest plots for selected Cox Proportional Hazards Models in KIRC and KIRP
(A) For the KIRC patients, metadata variables age, gender, and race were combined with the oncologic variable TreatmentType to create and analyze a multivariate Cox regression model. In addition to the number of patients associated with each variable (and variable subsets), the hazard ratio, 95% confidence interval for the hazard ratio, and the p-value associated with variable are shown. SLC22A2, SLC22A4, SLC22A6, SLC22A8, SLC22A11, SLC22A12, and SLC22A24 gene expression combined with these variables had the most significant association with overall survival. (B) For the KIRC patients, metadata variables age, gender, and race were combined with the oncologic variable PriMalig (prior malignancy) to create and analyze a multivariate Cox regression model. In addition to the number of patients associated with each variable (and variable subsets), the hazard ratio, 95% confidence interval for the hazard ratio, and the p-value associated with variable are shown. (C, D) Same as (A) for the KIRP patient’s metadata variables age, gender, and reported race and oncologic variable N (Lymph Node Involvement) (C) and oncologic variable T (Tumor Size and Progression) (D).

## Slide 3
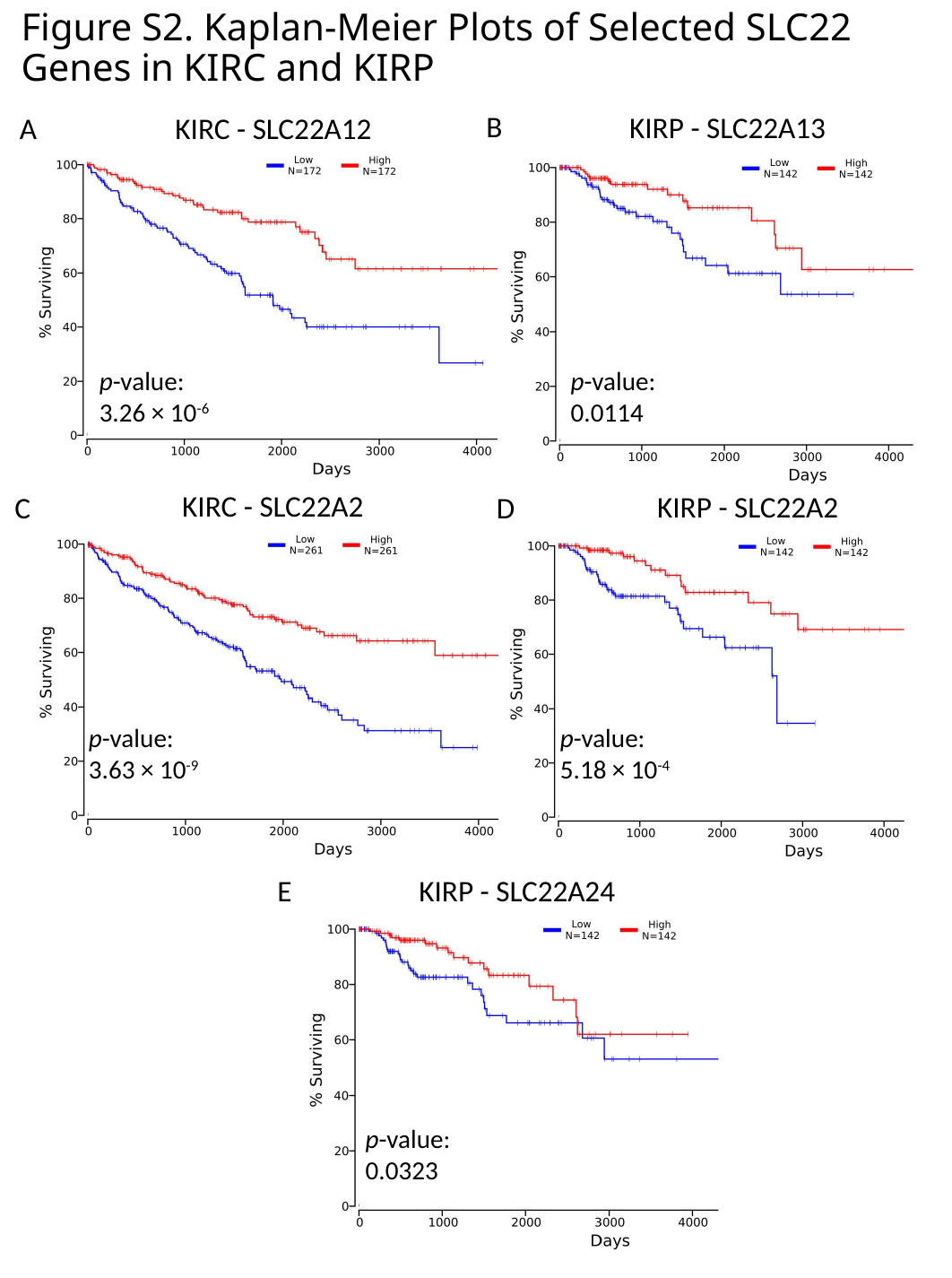

# Figure S2. Kaplan-Meier Plots of Selected SLC22 Genes in KIRC and KIRP
B
KIRP - SLC22A13
A
KIRC - SLC22A12
p-value: 3.26 × 10^-6^
p-value: 0.0114
KIRC - SLC22A2
KIRP - SLC22A2
C
D
p-value: 3.63 × 10^-9^
p-value:
5.18 × 10^-4^
E
KIRP - SLC22A24
p-value: 0.0323

## Slide 4
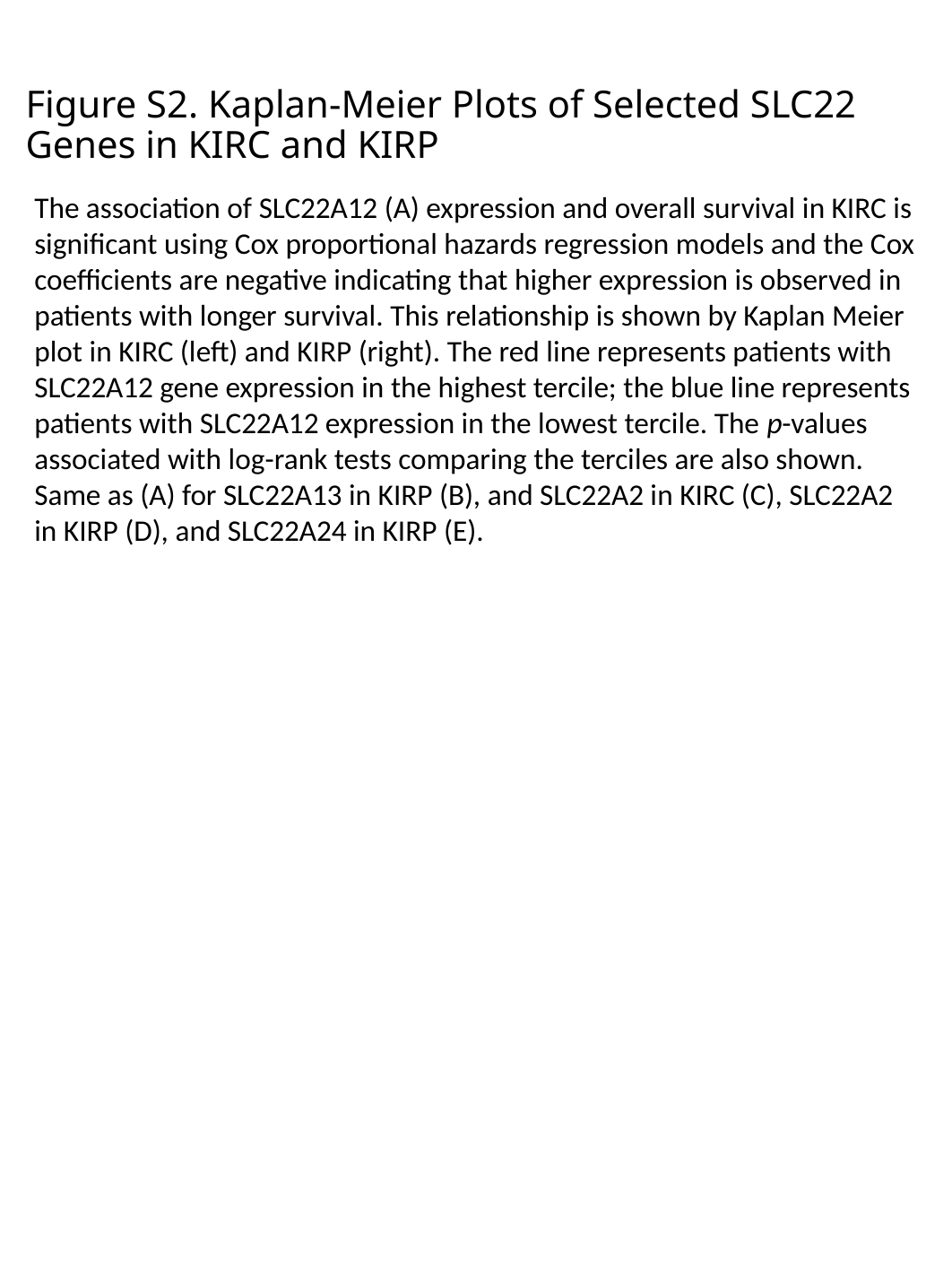

# Figure S2. Kaplan-Meier Plots of Selected SLC22 Genes in KIRC and KIRP
The association of SLC22A12 (A) expression and overall survival in KIRC is significant using Cox proportional hazards regression models and the Cox coefficients are negative indicating that higher expression is observed in patients with longer survival. This relationship is shown by Kaplan Meier plot in KIRC (left) and KIRP (right). The red line represents patients with SLC22A12 gene expression in the highest tercile; the blue line represents patients with SLC22A12 expression in the lowest tercile. The p-values associated with log-rank tests comparing the terciles are also shown. Same as (A) for SLC22A13 in KIRP (B), and SLC22A2 in KIRC (C), SLC22A2 in KIRP (D), and SLC22A24 in KIRP (E).

## Slide 5
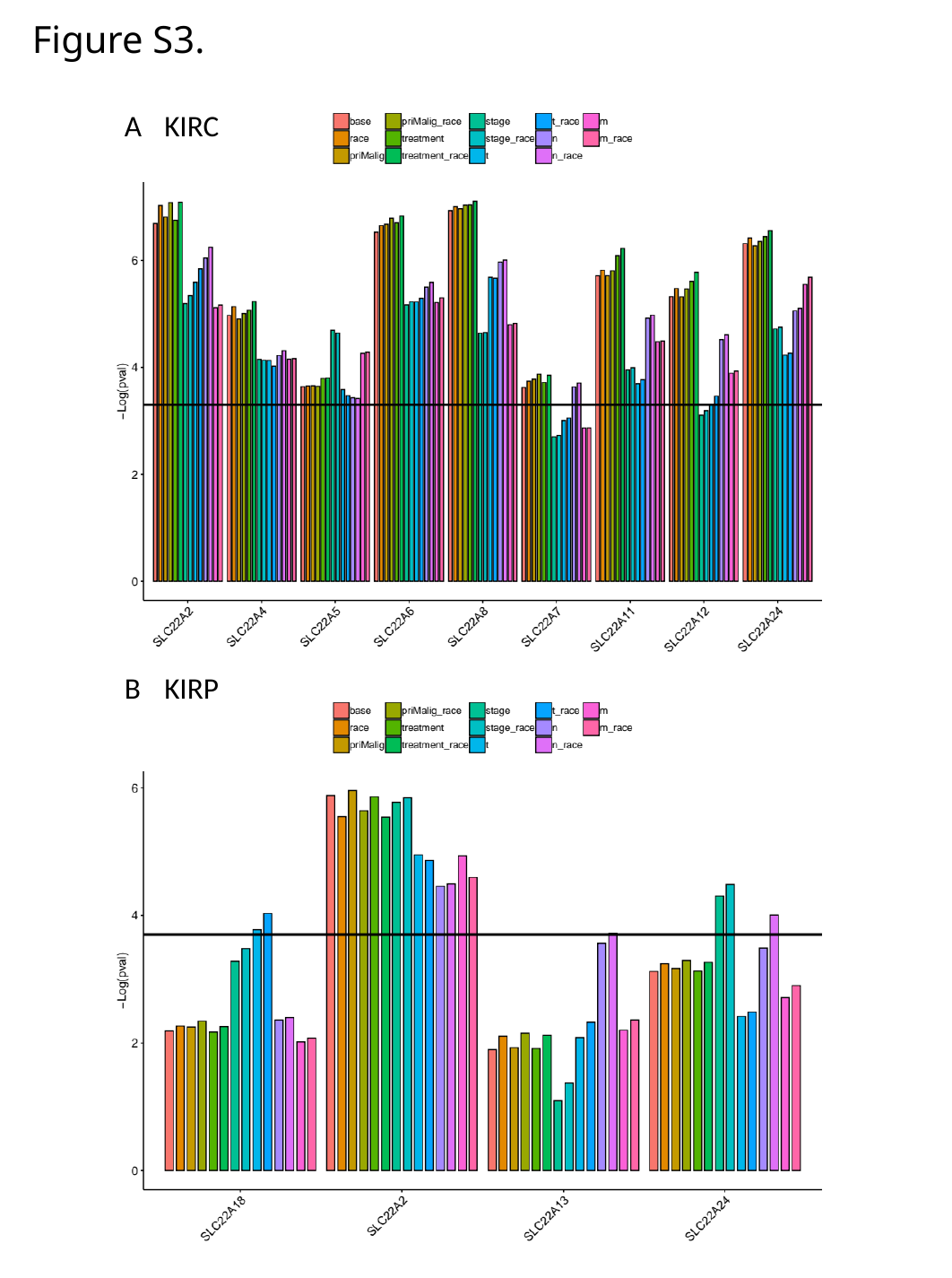

# Figure S3.
KIRC
A
KIRP
B

## Slide 6
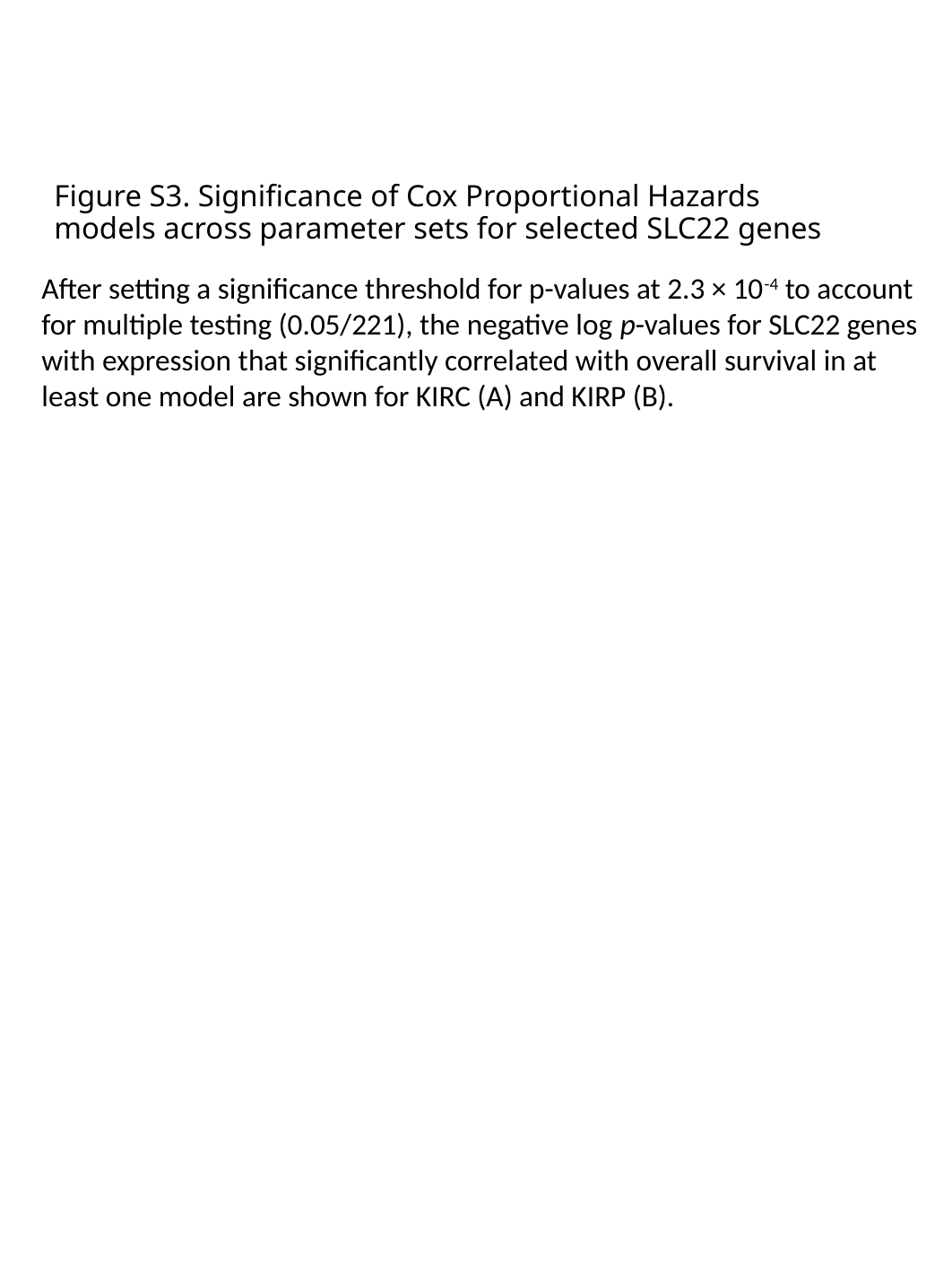

# Figure S3. Significance of Cox Proportional Hazards models across parameter sets for selected SLC22 genes
After setting a significance threshold for p-values at 2.3 × 10^-4^ to account for multiple testing (0.05/221), the negative log p-values for SLC22 genes with expression that significantly correlated with overall survival in at least one model are shown for KIRC (A) and KIRP (B).

## Slide 7
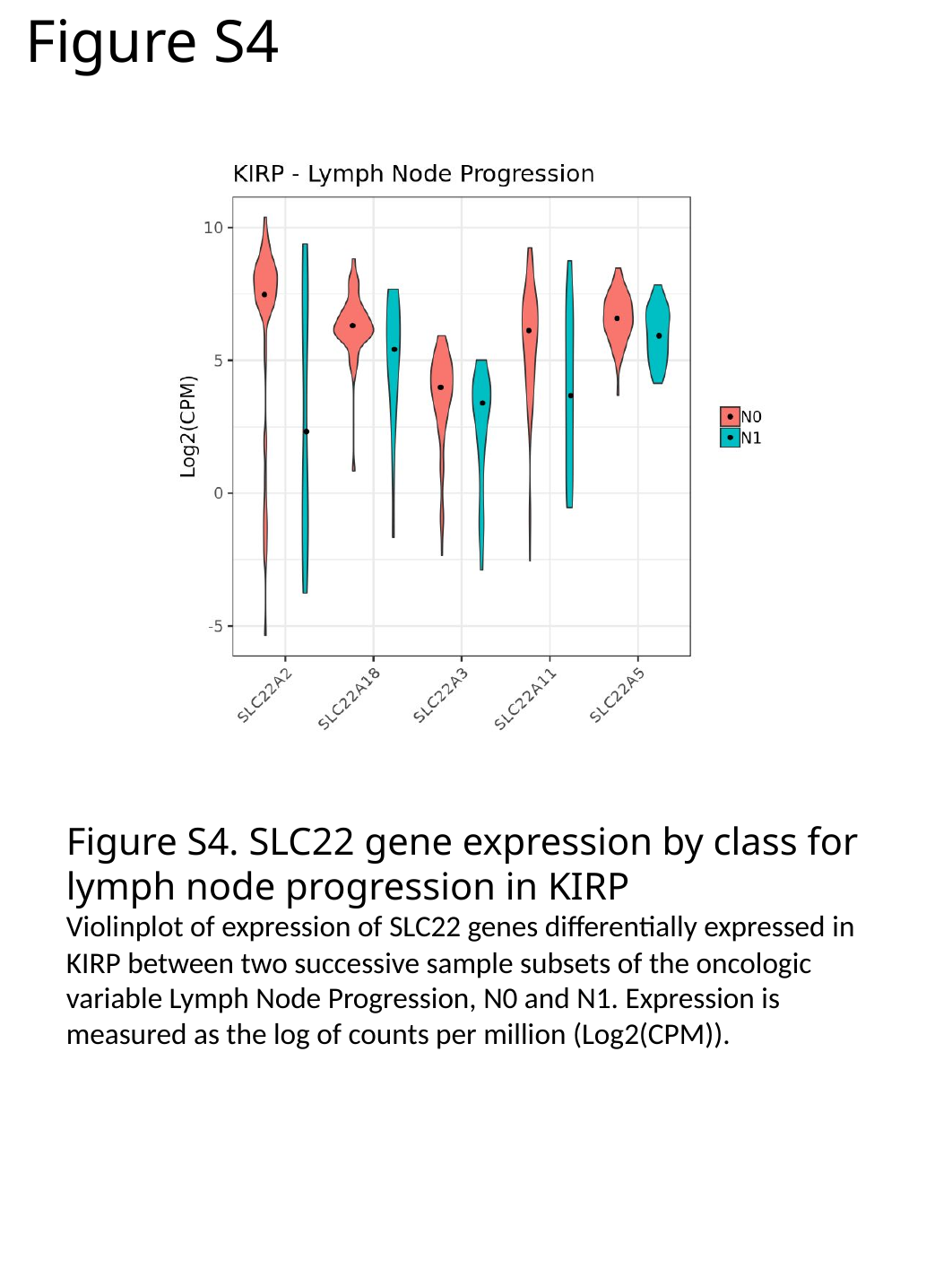

# Figure S4
Figure S4. SLC22 gene expression by class for lymph node progression in KIRP
Violinplot of expression of SLC22 genes differentially expressed in KIRP between two successive sample subsets of the oncologic variable Lymph Node Progression, N0 and N1. Expression is measured as the log of counts per million (Log2(CPM)).

## Slide 8
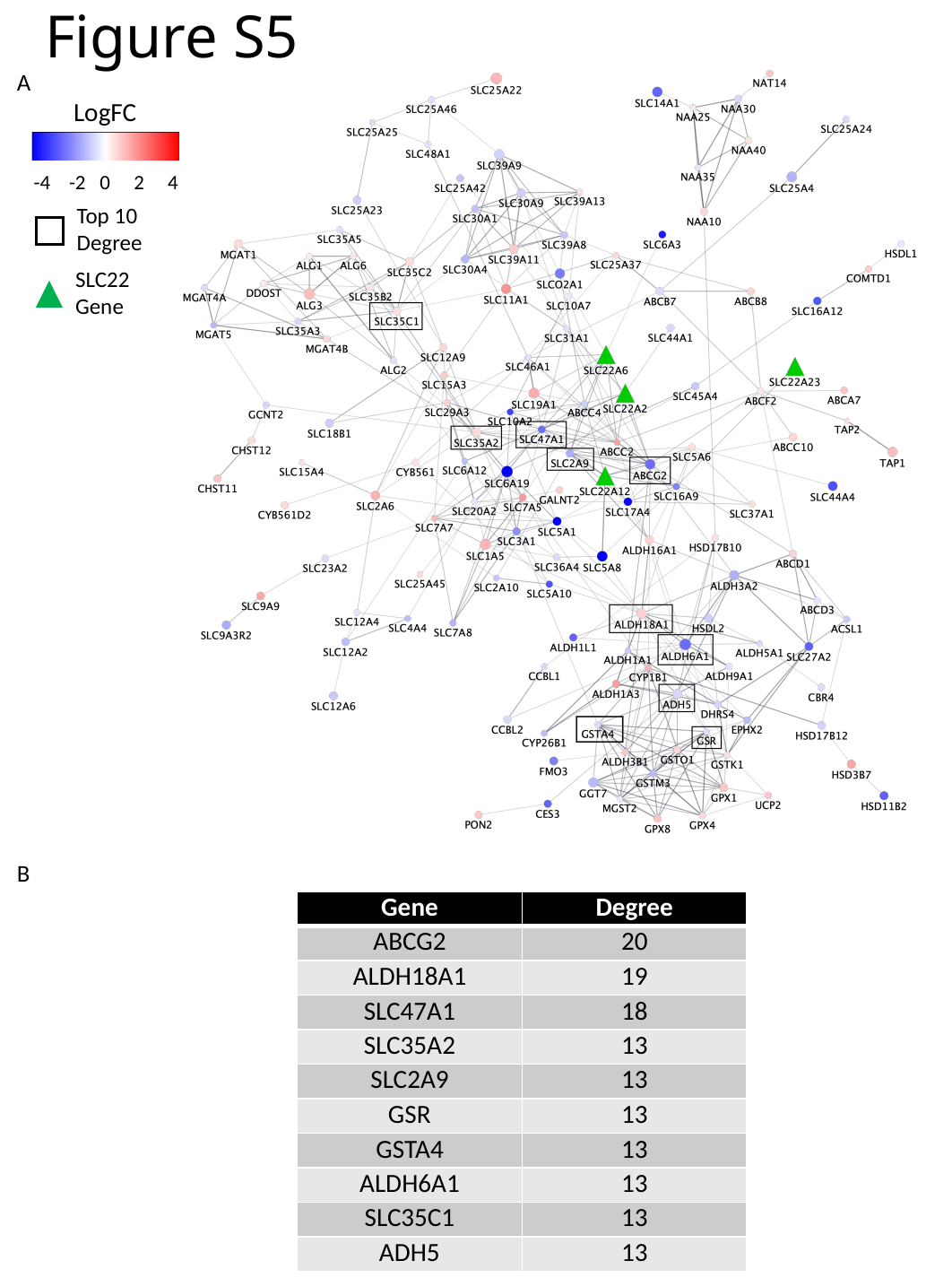

# Figure S5
A
LogFC
-4 -2 0 2 4
Top 10 Degree
SLC22 Gene
B
| Gene | Degree |
| --- | --- |
| ABCG2 | 20 |
| ALDH18A1 | 19 |
| SLC47A1 | 18 |
| SLC35A2 | 13 |
| SLC2A9 | 13 |
| GSR | 13 |
| GSTA4 | 13 |
| ALDH6A1 | 13 |
| SLC35C1 | 13 |
| ADH5 | 13 |

## Slide 9
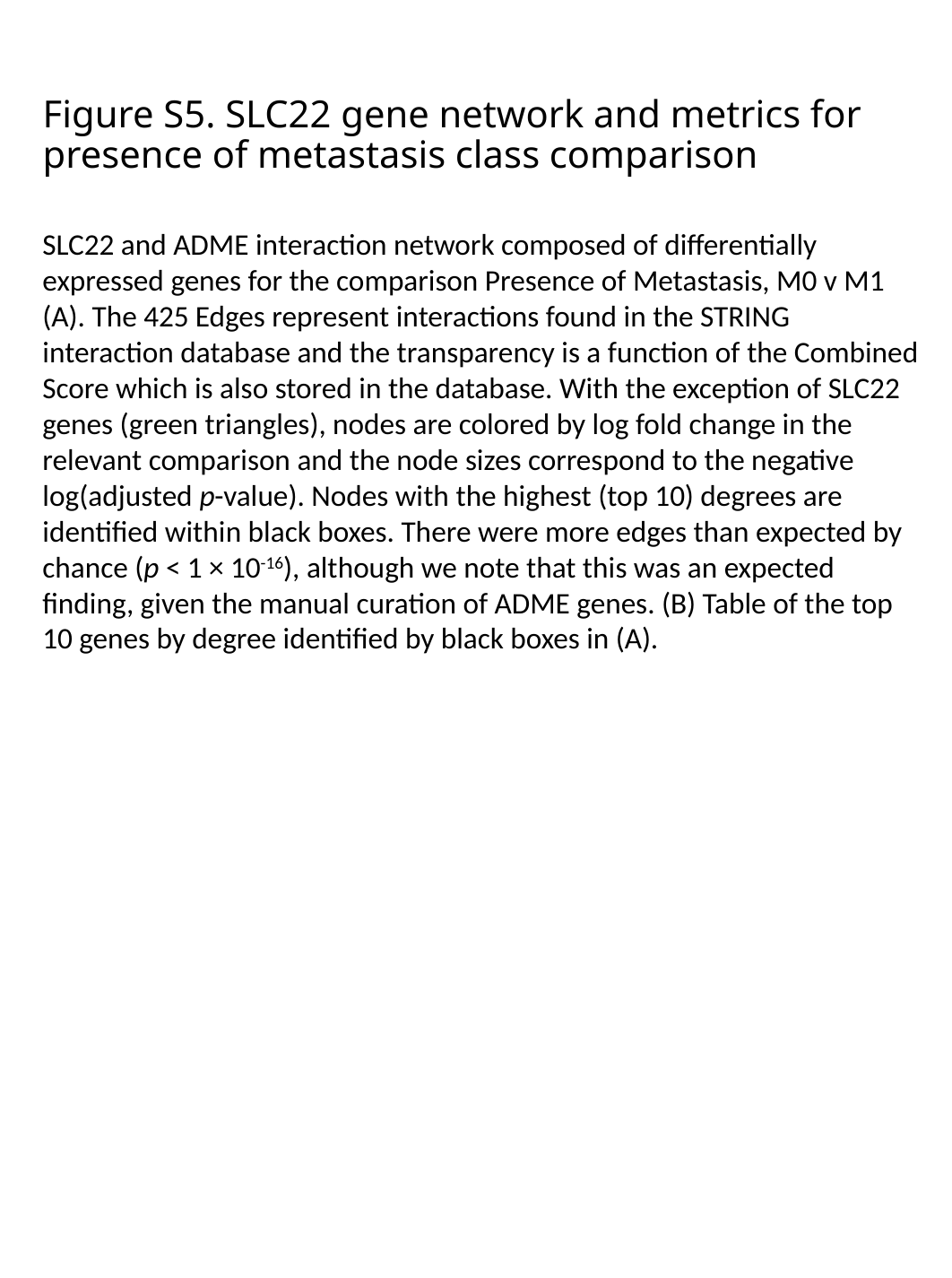

# Figure S5. SLC22 gene network and metrics for presence of metastasis class comparison
SLC22 and ADME interaction network composed of differentially expressed genes for the comparison Presence of Metastasis, M0 v M1 (A). The 425 Edges represent interactions found in the STRING interaction database and the transparency is a function of the Combined Score which is also stored in the database. With the exception of SLC22 genes (green triangles), nodes are colored by log fold change in the relevant comparison and the node sizes correspond to the negative log(adjusted p-value). Nodes with the highest (top 10) degrees are identified within black boxes. There were more edges than expected by chance (p < 1 × 10^-16^), although we note that this was an expected finding, given the manual curation of ADME genes. (B) Table of the top 10 genes by degree identified by black boxes in (A).

## Slide 10
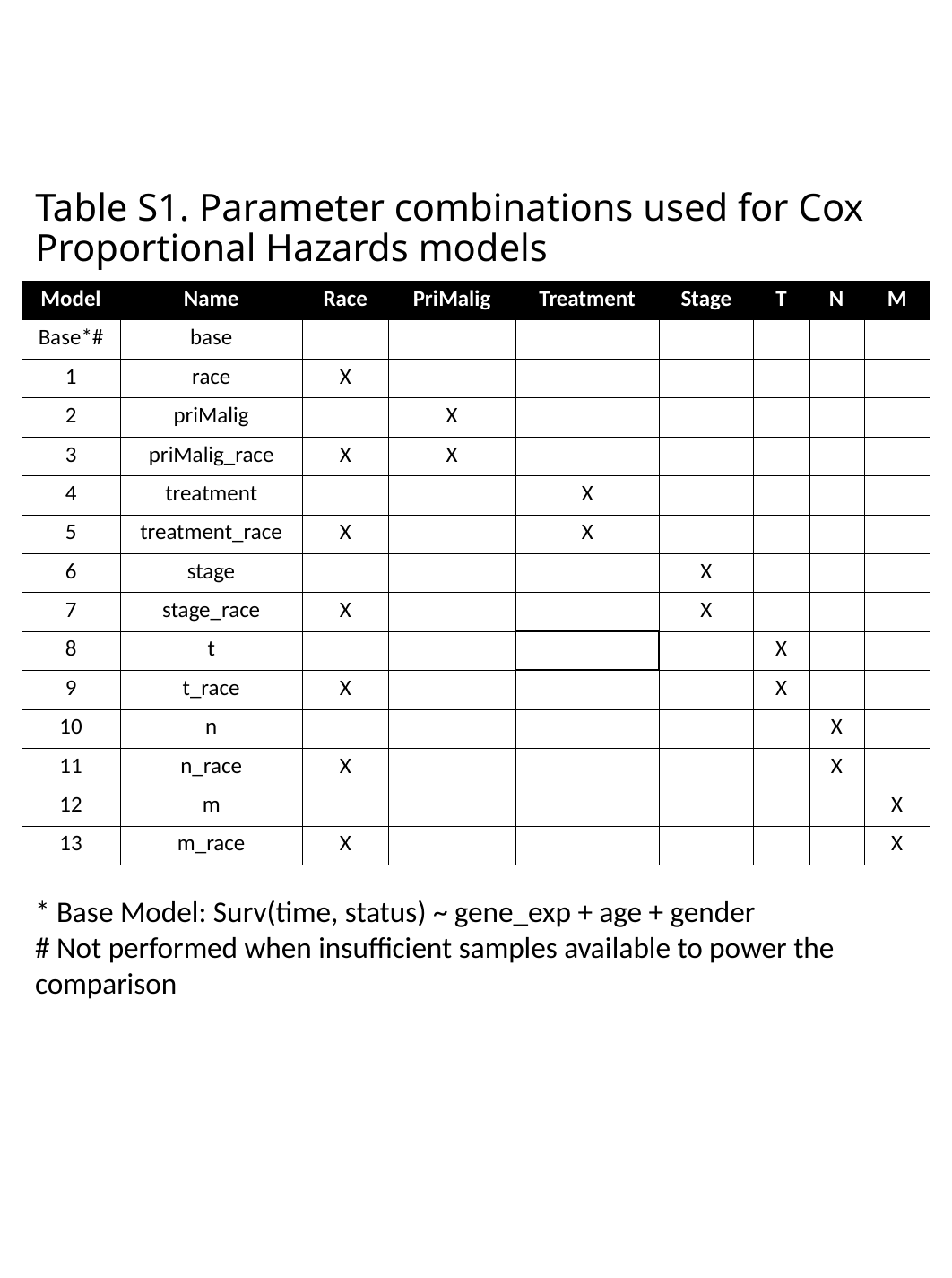

# Table S1. Parameter combinations used for Cox Proportional Hazards models
| Model | Name | Race | PriMalig | Treatment | Stage | T | N | M |
| --- | --- | --- | --- | --- | --- | --- | --- | --- |
| Base\*# | base | | | | | | | |
| 1 | race | X | | | | | | |
| 2 | priMalig | | X | | | | | |
| 3 | priMalig\_race | X | X | | | | | |
| 4 | treatment | | | X | | | | |
| 5 | treatment\_race | X | | X | | | | |
| 6 | stage | | | | X | | | |
| 7 | stage\_race | X | | | X | | | |
| 8 | t | | | | | X | | |
| 9 | t\_race | X | | | | X | | |
| 10 | n | | | | | | X | |
| 11 | n\_race | X | | | | | X | |
| 12 | m | | | | | | | X |
| 13 | m\_race | X | | | | | | X |
* Base Model: Surv(time, status) ~ gene_exp + age + gender
# Not performed when insufficient samples available to power the comparison

## Slide 11
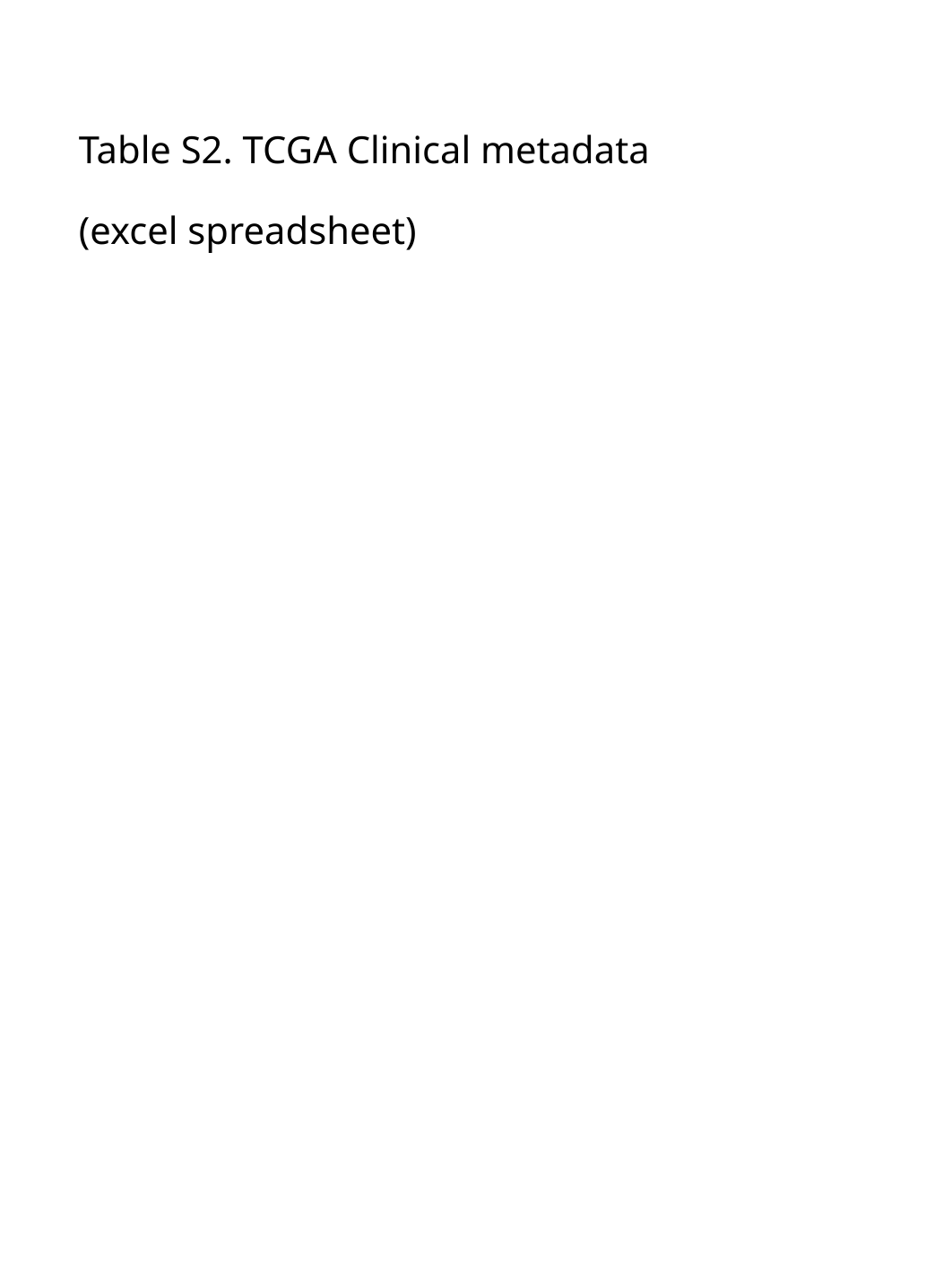

# Table S2. TCGA Clinical metadata (excel spreadsheet)

## Slide 12
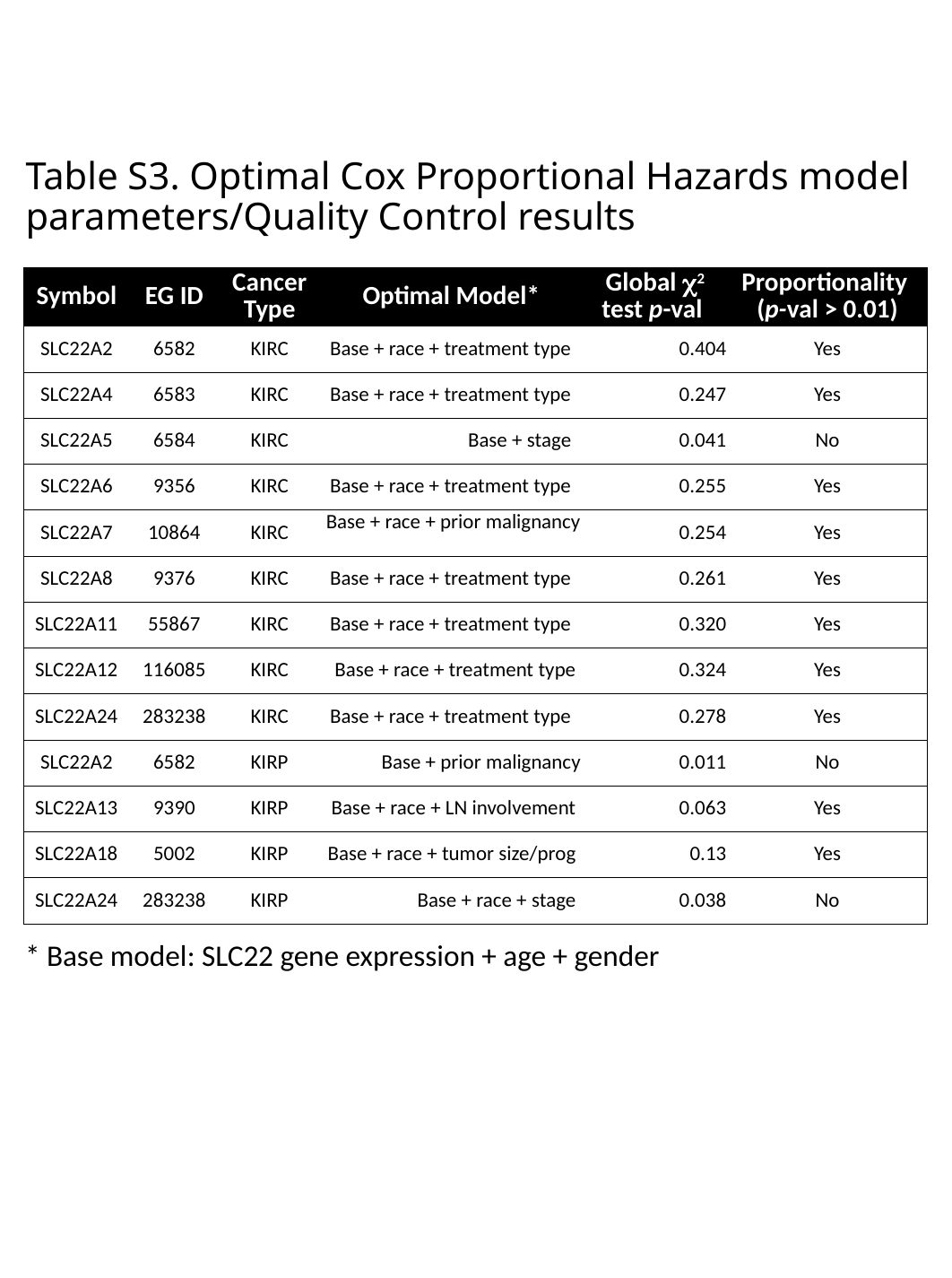

# Table S3. Optimal Cox Proportional Hazards model parameters/Quality Control results
| Symbol | EG ID | Cancer Type | Optimal Model\* | Global c2 test p-val | Proportionality (p-val > 0.01) |
| --- | --- | --- | --- | --- | --- |
| SLC22A2 | 6582 | KIRC | Base + race + treatment type | 0.404 | Yes |
| SLC22A4 | 6583 | KIRC | Base + race + treatment type | 0.247 | Yes |
| SLC22A5 | 6584 | KIRC | Base + stage | 0.041 | No |
| SLC22A6 | 9356 | KIRC | Base + race + treatment type | 0.255 | Yes |
| SLC22A7 | 10864 | KIRC | Base + race + prior malignancy | 0.254 | Yes |
| SLC22A8 | 9376 | KIRC | Base + race + treatment type | 0.261 | Yes |
| SLC22A11 | 55867 | KIRC | Base + race + treatment type | 0.320 | Yes |
| SLC22A12 | 116085 | KIRC | Base + race + treatment type | 0.324 | Yes |
| SLC22A24 | 283238 | KIRC | Base + race + treatment type | 0.278 | Yes |
| SLC22A2 | 6582 | KIRP | Base + prior malignancy | 0.011 | No |
| SLC22A13 | 9390 | KIRP | Base + race + LN involvement | 0.063 | Yes |
| SLC22A18 | 5002 | KIRP | Base + race + tumor size/prog | 0.13 | Yes |
| SLC22A24 | 283238 | KIRP | Base + race + stage | 0.038 | No |
* Base model: SLC22 gene expression + age + gender

## Slide 13
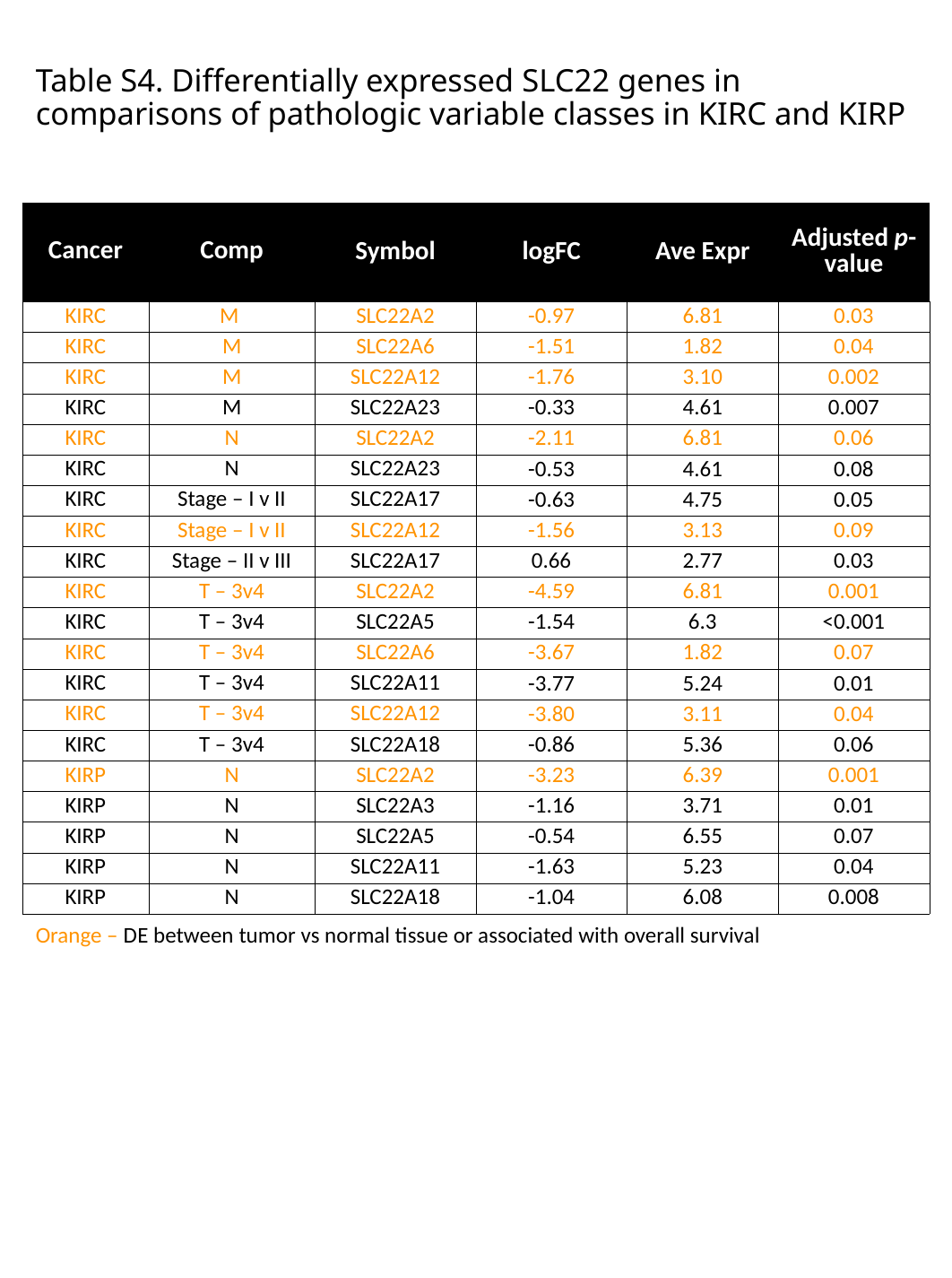

# Table S4. Differentially expressed SLC22 genes in comparisons of pathologic variable classes in KIRC and KIRP
| Cancer | Comp | Symbol | logFC | Ave Expr | Adjusted p-value |
| --- | --- | --- | --- | --- | --- |
| KIRC | M | SLC22A2 | -0.97 | 6.81 | 0.03 |
| KIRC | M | SLC22A6 | -1.51 | 1.82 | 0.04 |
| KIRC | M | SLC22A12 | -1.76 | 3.10 | 0.002 |
| KIRC | M | SLC22A23 | -0.33 | 4.61 | 0.007 |
| KIRC | N | SLC22A2 | -2.11 | 6.81 | 0.06 |
| KIRC | N | SLC22A23 | -0.53 | 4.61 | 0.08 |
| KIRC | Stage – I v II | SLC22A17 | -0.63 | 4.75 | 0.05 |
| KIRC | Stage – I v II | SLC22A12 | -1.56 | 3.13 | 0.09 |
| KIRC | Stage – II v III | SLC22A17 | 0.66 | 2.77 | 0.03 |
| KIRC | T – 3v4 | SLC22A2 | -4.59 | 6.81 | 0.001 |
| KIRC | T – 3v4 | SLC22A5 | -1.54 | 6.3 | <0.001 |
| KIRC | T – 3v4 | SLC22A6 | -3.67 | 1.82 | 0.07 |
| KIRC | T – 3v4 | SLC22A11 | -3.77 | 5.24 | 0.01 |
| KIRC | T – 3v4 | SLC22A12 | -3.80 | 3.11 | 0.04 |
| KIRC | T – 3v4 | SLC22A18 | -0.86 | 5.36 | 0.06 |
| KIRP | N | SLC22A2 | -3.23 | 6.39 | 0.001 |
| KIRP | N | SLC22A3 | -1.16 | 3.71 | 0.01 |
| KIRP | N | SLC22A5 | -0.54 | 6.55 | 0.07 |
| KIRP | N | SLC22A11 | -1.63 | 5.23 | 0.04 |
| KIRP | N | SLC22A18 | -1.04 | 6.08 | 0.008 |
Orange – DE between tumor vs normal tissue or associated with overall survival

## Slide 14
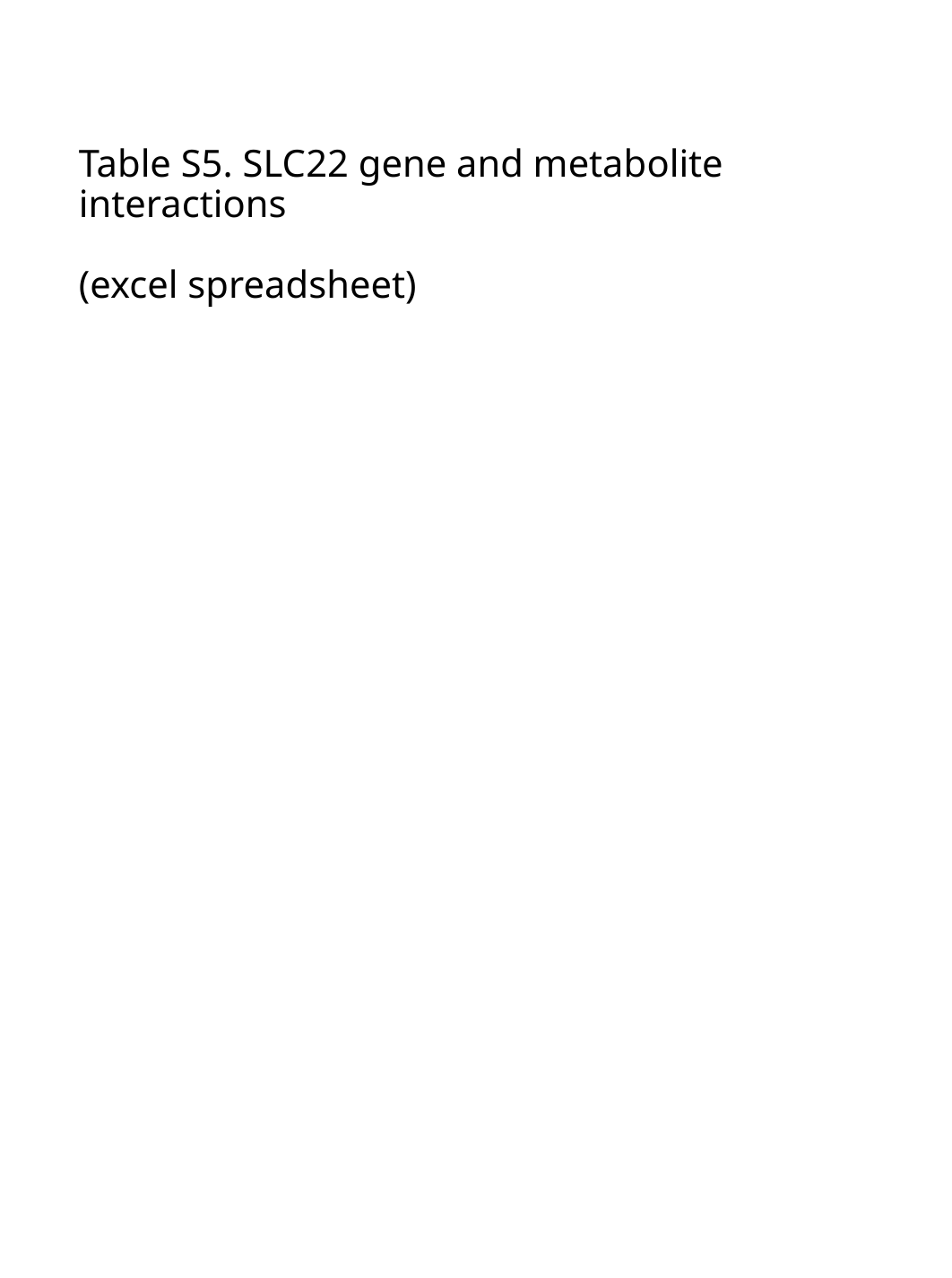

# Table S5. SLC22 gene and metabolite interactions (excel spreadsheet)

## Slide 15
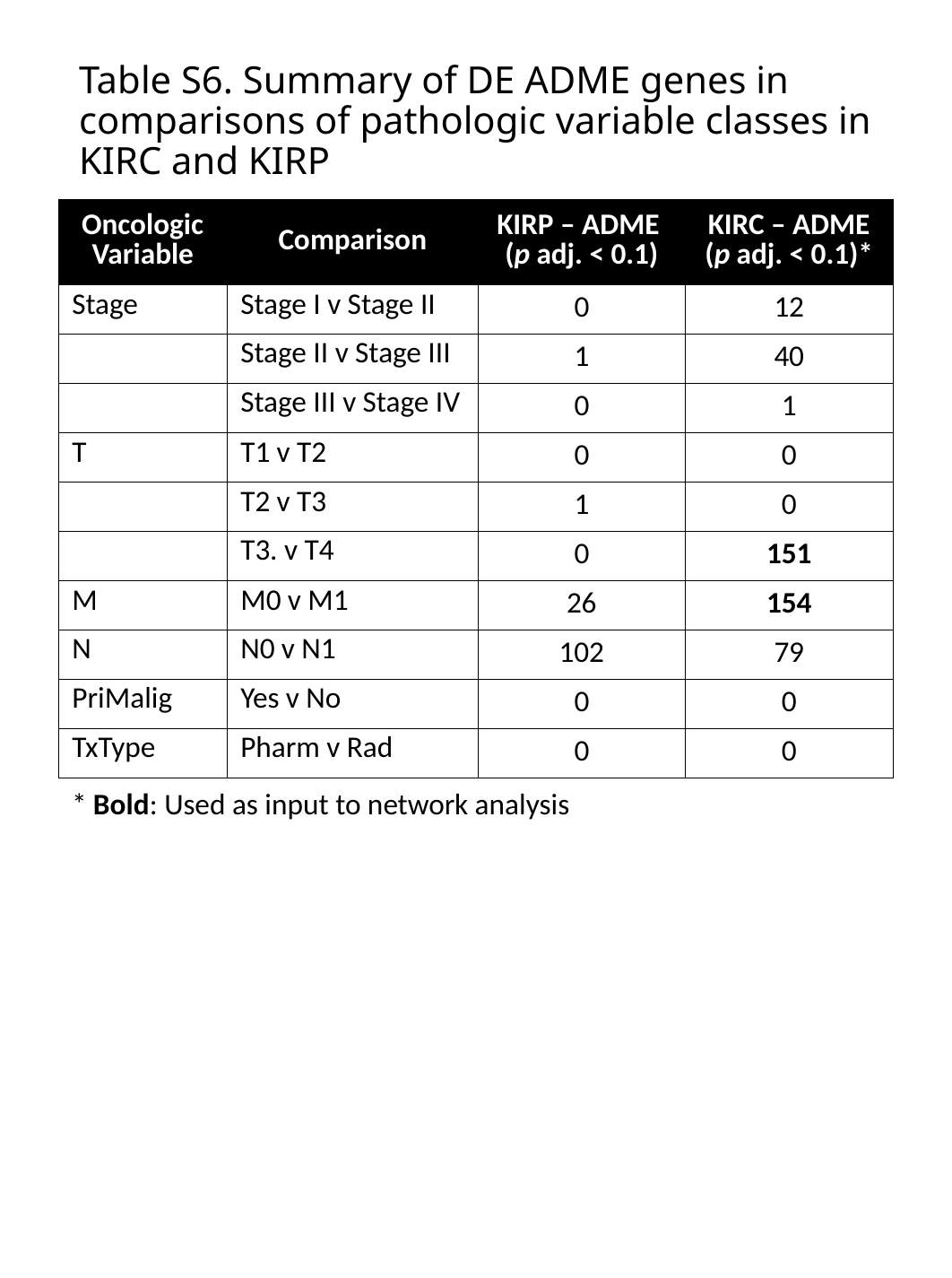

# Table S6. Summary of DE ADME genes in comparisons of pathologic variable classes in KIRC and KIRP
| Oncologic Variable | Comparison | KIRP – ADME (p adj. < 0.1) | KIRC – ADME (p adj. < 0.1)\* |
| --- | --- | --- | --- |
| Stage | Stage I v Stage II | 0 | 12 |
| | Stage II v Stage III | 1 | 40 |
| | Stage III v Stage IV | 0 | 1 |
| T | T1 v T2 | 0 | 0 |
| | T2 v T3 | 1 | 0 |
| | T3. v T4 | 0 | 151 |
| M | M0 v M1 | 26 | 154 |
| N | N0 v N1 | 102 | 79 |
| PriMalig | Yes v No | 0 | 0 |
| TxType | Pharm v Rad | 0 | 0 |
* Bold: Used as input to network analysis

## Slide 16
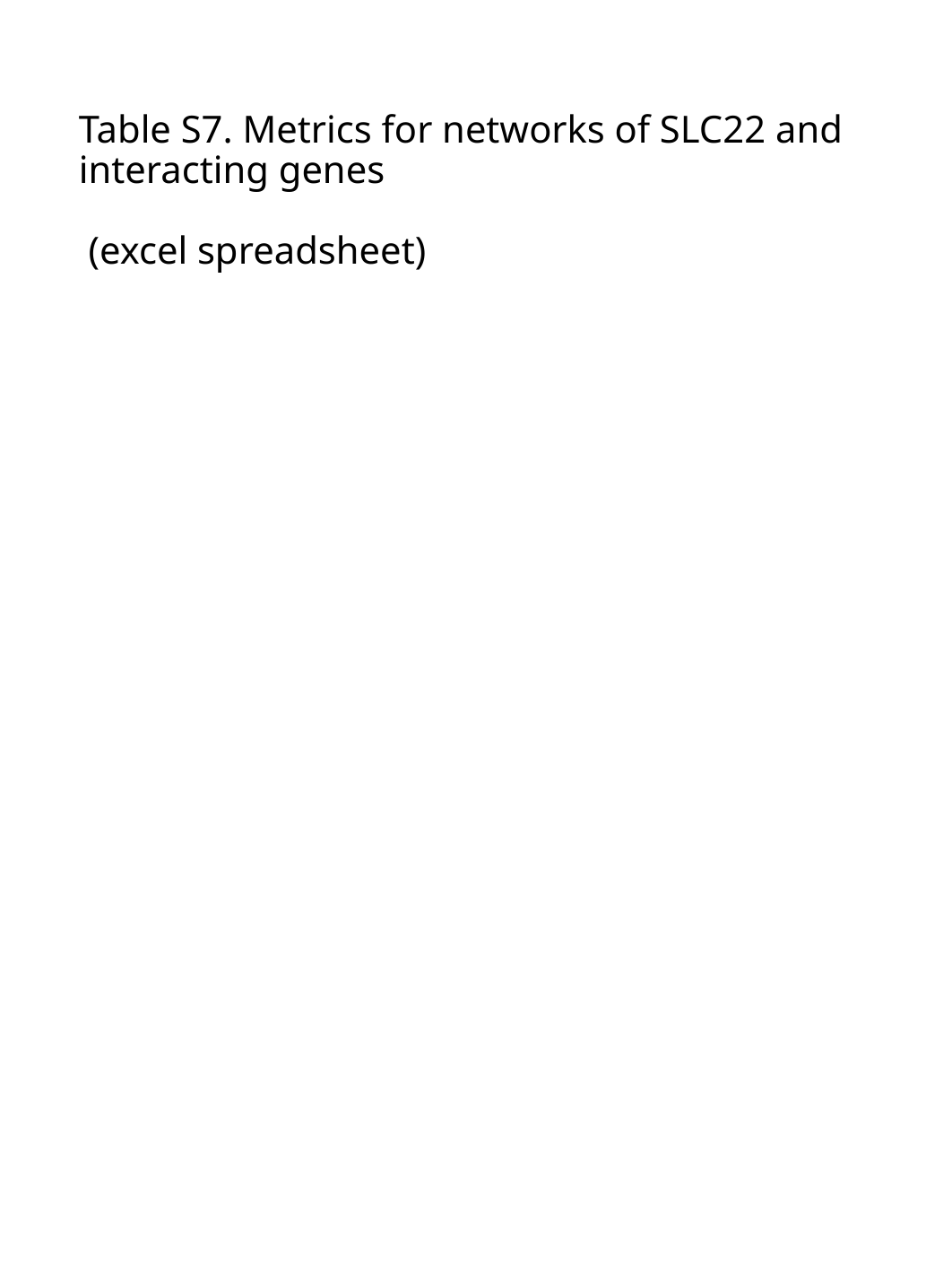

# Table S7. Metrics for networks of SLC22 and interacting genes (excel spreadsheet)
